# Obstetric violence and associated factors among women during facility based childbirth at Gedeo Zone, South Ethiopia

**DOI:** 10.1186/s12884-022-04895-6

**Published:** 2022-07-14

**Authors:** Wondwosen Molla, Aregahegn Wudneh, Ruth Tilahun

**Affiliations:** Department of Midwifery, Dilla University, Dilla, Ethiopia

**Keywords:** Obstetric violence, Childbirth, Ethiopia

## Abstract

**Introduction:**

Obstetric violence is a specific form of violence against women that violates their human rights. Conducted by obstetric care providers regarding the body and reproductive processes of the woman, being characterized by dehumanized assistance, abuse of interventionist actions, medicalization, and reversion of the process from natural to pathological.

**Objective:**

To assess the magnitude of obstetric violence and associated factors among women during childbirth in Gedeo Zone, South Ethiopia.

**Method:**

Community based cross-sectional study was conducted among randomly selected 661 mothers in Gedeo Zone, South Ethiopia, from May 1 to May 30 2020. Multi-stage sampling technique was used to get a total of 661 mothers from their kebeles. Data was collected by using face-to--to-face interview with a structured questionnaire and in-depth interview was also employed. Data entry and analysis was done by Epi data version 3.1 and SPSS 23.0 statistical software. Bivariate and multivariable logistic regression models were used to determine the important predictors of obstetric violence. Association between outcome and independent variables was presented by adjusted odds ratio with 95% CI.

**Results:**

From the total of 661 mothers, about 79.7% (527) of mothers experienced obstetric violence with 95% CI (76.9–82.8). educational status (AOR = 2.2573, 95%CI = 1.44,3.54), ANC utilization (AOR = 2.365, 95%CI = 1.62–3.21), duration of stay (AOR = 0.5367,95%CI = 0.28,0.86)), and facing complication during labor and delivery (AOR = 3.1382, 95%CI = 2.34,5.17) were the major factors associated with obstetric violence.

**Conclusion:**

The magnitude of obstetric violence was high. Non dignified care and non-consented care was the most common form of obstetric violence which may lead a woman to choose for home delivery instead of health facility care, this in turn leads to a great increase in maternal morbidity and mortality as supported by qualitative approach of the study.

## Introduction

Obstetric violence (OV) is a specific form of violence against women that violates their human rights. As stated in organic Law on Women’s Rights in other developed countries, it is a violation of women’s right to have a violation free life, steered by the obstetric care providers regarding the body and the reproductive processes of the woman [1, 2]; which incorporates dehumanized assistance, abuse of interventionist actions, medicalization and reversion of the process from natural to pathological, that results on loss of their right to decide about their own body and negative impact on quality of women’s life [3, 4]. The term obstetric violence is clearly stated in the law of Venezuela as a punishable act of breach of women’s right to ‘life free of violence. Obstetric violence is clearly defined in Venezuelan law as a punishable violation of women’s right to a “life free of violence.” One of the relevant rights of women specified by law is the right to life, which is one of the components of human rights [5, 6].

The WHO statement on obstetric violence addresses OV because it not only violates women’s rights to respectful care, but also threatens their right to life as a violation of human rights, health, bodily integrity, and freedom from discrimination [7–9].

The term OV is also addressed in many literatures relating to the concept of human rights because every woman is equally free to exercise her human rights and freedoms, which include: the right to life; the right to physical, psychological, and moral integrity; the right to freedom and personal safety; the right not to be tortured; and the right to have her dignity respected and her family protected; The right to equality of protection from the law and by the law [9, 10].

Obstetric violence persists in various forms around the world (verbal violence, physical violence, sexual violence, social discrimination, neglect of care, and inappropriate use of procedures and skills), despite the fact that it is unacceptable to health care providers and society [1, 4, 11–13]. It happens in both public and private health care facilities during obstetric care, and it causes physical or psychological harm to a woman during pregnancy, birth, and puerperium [2, 14, 15] because OV is a new term, it is not recognized as a human rights violation [6, 11].

According to various literatures, obstetric care providers provided care with no scientific evidence to support its use. Interventions such as avoiding a woman’s concerns during childbirth, frequent vaginal palpations, using oxytocin, shaving the vulva, or applying episiotomy are routinely performed, and many are not even documented in women’s medical records [11, 16].

According to a study conducted in Spain, the magnitude of the problem was alarming, with nearly half of women responding negatively if they had received unnecessary and/or painful procedures while giving birth [11], and hospitals being identified as the area with the most obstetric violence for the various studied variables. Findings such as lack of information and informed consent (74.2%) and criticism of infantile behavior and treatment (87.6%) indicate that women experienced more obstetric violence during childbirth [17].

Preventing and reducing violence against women and maternal mortality are sustainable development goals (SDGs) [18, 19]. Every day, approximately 830 women die around the world, with developing countries accounting for 99% of maternal deaths [20]. Many countries, including Ethiopia, have bet on improved access to health care facilities as a means to reduce maternal mortality. However, maternal mortality is only a small proportion of the global burden of the maternal morbidity spectrum. This is because for one maternal death there are many women affected by severe acute maternal morbidity (SAMM) during pregnancy, childbirth, and the postpartum period, this is might be occurred due to obstetric violence [21, 22]. WHO calls for action and research to prevent and eliminate this invisible wound and human rights issue (obstetric violence) [23]. Therefore, this study is helpful to identify the magnitude of obstetric violence and associated factors in Ethiopia where the problem is not well addressed.

## Methods and materials

### Study area& period

The study was conducted in Gedeo Zone, South Ethiopia from January1 to February 15, 2020 GC. Gedeo zone is located in Southern 360Km far from Addis Ababa, capital city of Ethiopia. There are six districts, two city administrations with 31 urban and 133 rural kebeles (the smallest administration unit) with one referral hospital, three district hospitals, 38 health centers, five NGO,36 private clinics and 47 drug vendors in the zone [24].

Study design; Community based cross-sectional study supplemented by qualitative methods was conducted.

Study population; all mothers who gave birth in a health facility within the last year prior to data collection and who had lived in the Gedeo zone for at least 6 months were included in this study. This study excluded mothers who were critically ill and unable to communicate at the time of data collection.

### Sample size determination

The sample size was calculated using the single population proportion formula (n = (Z/2)2p (1p)/d2) with 0.05 margins of error at the 95% confidence level (CI), 50% was used as a proportion of obstetrical violence because no similar study was conducted in Ethiopia. The sample size was then multiplied by 1.5 to account for the design effect, and for possible none response rate during the study sample size was increased by 10% to: *n* = 674.

For the qualitative part, Purposive sampling was used to recruit study participants for the qualitative portion of the study based on the concept of data saturation. In-depth interviews were conducted with a total of 11 study participants with the aim of getting a greater depth of response about OV among purposely selected key informants from health care facilities, 4 midwifery (MCH ward head), 3 community leaders, and 4 mothers who used maternity care but were not included in face-to-face interviews with structured questionnaires for the quantitative part.

### Sampling techniques and procedures

Multi-stage sampling technique was used. Initially, out of 6 districts of the Zone, three districts were selected by using simple random sampling techniques (lottery method). From a total of 69 kebeles (the smallest administration unit) at the selected districts; eleven out of 31, ten out of 29, and twelve out of 33 kebeles were selected by using lottery methods from Yirgacheffie, Bule and Wonago districts respectively. Then the census was conducted to identify the number of women who gave birth in the last one year at the health facility that avail in the selected kebeles. Based on the census, a total of 2246 women were identified. Of those, 840 were in Yirgacheffie district, 650 were in Bule, and 756 were in Wonago district.

A simple random sampling technique was used to get a total of 674 women out of 2246 women who identified and registered during census. Households were sampling units for this study and the final sample size was allocated proportionally for each kebele based on the number of women. Therefore, 252 mothers from Yirgacheffie district, 195 from Bule, and 224 from Wonago district were allocated to participate in the study. Then the study households were selected from each kebele through a simple random sampling technique by using a computer-generated random number starting from kebele one from a random start point after developing the sampling frame having a list of individual’s house number which was given during census. One mother per household was interviewed. When two or more eligible pregnant women were found in one household, only one was interviewed by using the lottery method.

### Data collection methods and procedures

For the quantitative part, a standard tool was adapted, which was prepared in the English language, and then translated to Amharic and Gedieo Uoffa local languages and back-translated to English language was done by an independent translator for consistency. Pre-test was done on 10% of the total participants (67 women) in Gongua town near to the study area. During the pretest, the questionnaire was assessed for its clarity, readability, comprehensiveness, accuracy, and optimal time for completing the interview. Based on the results of the pretest, modifications and corrections were made.

This tool was aimed to measure OV by using standard performance indicators and their respective verification criteria. Categories of OV include Physical abuse, Non-confidential care, Non-consented care, Non-dignified care, Discrimination luck of equitable care, Abandonment or denial of care, Detention in facilities, and Sexual violence. Accordingly, those women who replied ‘yes’ to at least one form of OV had been labeled to be subjected to OV. A total of 25 verification criteria were used to measure OV.

Internal consistency/reliability of the item was checked by computing Cronbach’s alpha. The value of Cronbach’s alpha was within the range of the normal value.

Data was collected through face-to--to-face interviews moved from house to house by the data collectors after written consent was obtained from the respondents. The data collection process was conducted individually at a convenient location of the respondents.

For the qualitative part, semi-structured guiding question with five items was prepared first in English. With some modifications, a topic guide was produced in Amharic to facilities in-depth interviews. To begin the interview, the following broad data-generating question was used: (1) Are you familiar with obstetric violence? (2) What types of obstetric violence are the most common? (3) did any of your neighbors or relatives tell you about their violent experiences? (4) Do you have any experience with obstetrical violence? (5) What is the most common cause of obstetric violence? During in-depth interviews and key informant discussions, audiotape recorders and notes were used to document the data. Audiotaped interviews lasted about 30 to 40 min and were usually conducted in the homes of the participants’ preference.

### Data analysis and interpretation

Data were checked for completeness, edited, and coded. The verification criteria were dichotomized responses, “Yes” or “No” to identify reported events of obstetric violence. For categories of obstetric violence with more than one verification criterion, a woman was labeled as “obstetric violence in the respective category” if she reported “Yes” to at least one of the verification criteria during childbirth. On the other hand, a mother who identified as having faced obstetric violence in at least one of the seven categories was considered “obstetric violence”. The data was entered by using Epi data version 3.1 software and then exported to SPSS version 23.0 statistical software for analysis. Descriptive statistics such as mean, median, frequency, and percentage were used. Bivariate logistic regression analysis was used to identify candidate variables for multivariable logistic regression. Variables that had *P*- value less than 0.25 were candidates for multivariable logistic regression. Multivariable analysis was used to determine the factors that are independently associated with obstetric violence. The odds ratio at 95 CI was computed to measure the strength of the association between the outcome and explanatory variables. Variables that had *P* value less than 0.05 was considered as statistically significant. Model fitness test was checked by Hosmer and Lemeshow test. The value was 0.914. Finally, the results were presented in the form of texts, tables, and graphs.

For qualitative data, the interviews were professionally transcribed and checked by the investigators for accuracy. Each interview was coded and continued until no additional participants were obtained after data saturation. The open code software was used to analyze the qualitative data. The data was transcribed verbatim and translated from Amharic to the English language by the research team and language exports independently. The analysis was done by the team members repeatedly read each of these transcripts, identified statements and quotes. Data were extracted and described in narratives thematically. Three of the authors have participated in the thematic analysis. Finally, the ideas were triangulated with the quantitative results.

### Data quality

To keep the data quality, standard, questionnaire was adapted. The data collectors and supervisors were trained for 02 days on the aims of the research, content of the questionnaire, and how to conduct an interview to increase their performance in the activities. Pretest was conducted on 10% of study participants with participants from the other side of the study area. All interviews were collected at the residences of the study participants. Empty or closed houses during the day of visit were revisited two times to preserve the required sample size. The Collected data were checked every day by supervisor and principal investigators for its completeness and consistency. All questionnaires were kept under lock and key for security and confidentiality of gained information.

## Result

### Socio-demographic characteristics

A total of 661 mothers were participated in the study, making a response rate of 98.1%. Majority of the respondents 252 (38.1%) were between 25 and 29 years age group with a mean age of 30.8 (SD ± 4.6). More than half of the respondents 390(59%) were from Gedeo ethnic group, 239 (36,2%) were followers of protestant religion. Regarding the marital status of the mothers, 531(80.3%) of them were married, 288 (43.6%) were house wives, 355 (53.7%) had a monthly family income less than 2500 Ethiopian birr. More than 455 (68.8%) of the respondents have no ability to pay for any medical service. The educational status of the mothers showed that 153 (23.1%) were unable to read and write and those who achieved educational level of college and above were 102 (15.4%) as see in Table 1.

**Table 1:** Socio demographic characteristics of the mother in Gedeo zone, south, Ethiopia, 2020(*n* = 661)

Variable	Frequency(***N*** = 661)	Percent (%)
**Age**		
16–19	19	2.9
20–24	52	7.9
25–29	252	38.1
30–34	232	35.1
≥ 35	106	16
**Occupation**		
House wife	288	43.6
Government employee	150	22.7
Private business	184	27.8
Other	39	5.9
**Educational background of mothers**		
Illiterate	153	23.1
Read and write	86	13
Primary	117	17.7
Secondary	203	30.7
Collage and above	102	15.4
**Educational background of husbands (587)**		
No formal education	92	15.7
Read and write	94	16
Primary	163	27.8
Secondary	107	18.2
Collage and above	131	22.3
**Religion**		
protestant	270	40.8
Orthodox	239	36.2
Muslim	152	23
**Marital status**		
Married	587	88.8
Single	16	2.4
Divorced	48	7.3
Widowed	10	1.5
**Ethnicity**		
Gedeo	390	59
Oromo	123	18.6
Amhara	35	5.3
Gurage	55	8.3
Others	58	8.8
**Income**		
Less than 2500	355	53.7
Greater than 2500	306	46.3
**Ability to pay for delivery**		
Yes	206	31.2
No	455	68.8

### Obstetrical characteristics of the mother

From the total of 661 mothers, most of 351(53.1%) of the respondents were para two and three followed by 158(23.9%) para one while the rest 152(23%) were para four and above. From the overall mothers who were participated in the study, 458(69.3%) had a history of ANC follow-up for the most recent child birth. From those who have a history of ANC follow-up, 276(60.2) of them had one up to three number of ANC follow-up and nearly half 225 (49.1%) of the mothers were received ANC follow-up by midwifes. Majority 383 (83.6%) of mothers who received ANC service were seen at public health facilities, primarily at health centres. From the total of 661 mothers, the majority 586(88.7%) of the respondents were given birth at public health facilities while the rest 75(11.3%) were at private health facilities. The dominant birth attendants as a report of the mothers were midwifes which was 446(67.5%) followed by 123 (18.6%), 92(13.9%) were Nurses and Doctors respectively. Moreover, most 411(62.2%) of the mothers gave their recent birth through spontaneous vaginal delivery (SVD). Around 108(16.3%) of the mother and 67 (10.2%) of the children were developed complications at the time of delivery **(see** Table 2 at the end of the document for more information).

**Table 2:** Obstetrical characteristics of the mothers in Gedeo Zone, South Ethiopia, 2020 (*n* = 661)

Type of variables	Frequency	Percent (%)
**Maternal ANC follow up (661)**		
Yes	458	69.3
No	203	30.7
**Place of receiving ANC(*N*** **= 458)**		
Health centre	227	49.6
District hospital	98	21.4
Referral hospital	58	12.6
Private institutions	75	16.4
**Profession of HCP conducting ANC (458)**		
Doctor	95	20.8
Nurse	138	30.1
Midwife	225	49.1
**Number of ANC (*N*** **= 458)**		
< 4	276	60.2
≥ 4	182	39.8
**Total births (661)**		
1	158	23.9
2–3	351	53.1
≥ 4	152	23
**Type of health facility (place of Birth) (661)**		
Public	586	88.7
Private	75	11.3
**Profession of HCP conducting delivery (661)**		
Nurse	123	18.6
Midwife	446	67.5
Doctor	92	13.9
**Sex of HCP conducting delivery (661)**		
Male	213	32.3
Female	448	67.7
**Mode of recent delivery (661)**		
SVD	436	65.9
AVD	225	34.1
**Number of days stayed (661)**		
1–2 day	562	85
≥ 3 day	99	15
**facing complication during delivery (661)**		
Yes, for my self	108	16.3
Yes, for child	67	10.2
Yes, for both	28	4.2
No at all	458	69.3
**Preferred birth position (661)**		
Kneeling	212	32.1
Squatting	128	19.3
Lithotomic	321	48.6
**Time of delivery (661)**		
Day	275	41.6
Night	386	58.4

*HCP* Health Care Providers, *SVD* Spontaneous Vaginal Delivery, *AVD* Assisted Vaginal Delivery

### Magnitude of obstetric violence

**Fig. 1:**
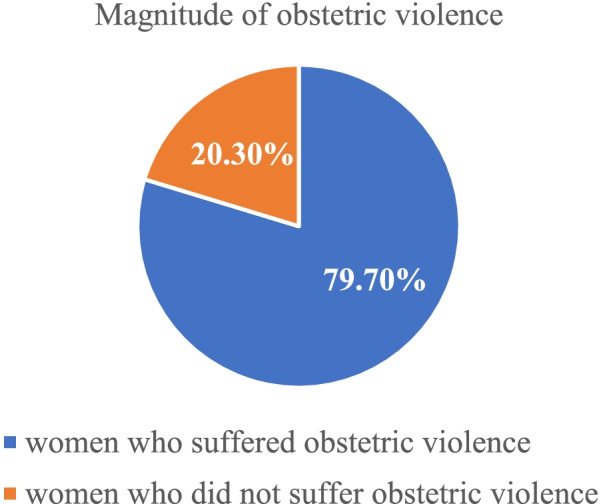
Magnitude of obstetric violence during facility-based child birth in Gedeo Zone, southern Ethiopia, 2020

From the total of 661 participants, majority 527(79.7%) of mothers experienced obstetric violence with 95% CI (76.9–82.8), while only 134(20.3%) of mothers didn’t experienced any form of obstetric violence, as see in Fig. 1**.**

### Forms of obstetric violence

**Fig. 2:**
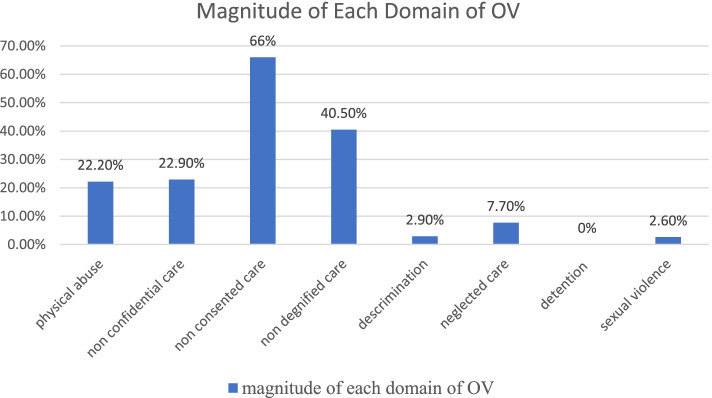
Magnitude of obstetric violence with category during facility-based childbirth in Gedeo Zone, Southern Ethiopia, 2020

Based on the verification criteria for categories of obstetric violence, mothers who experienced at least one form of the criteria were counted. The major form of obstetric violence experienced was unconsented care in which 436(66%) of mothers reported non-consented care. The second commonly reported type of obstetric violence as non-dignified care in which 40.5% of participants was reported. Demonstrating caring culturally in an appropriate way in which 30.1% of mothers were treated in a culturally inappropriate manner. The least commonly reported domain of obstetric violation was sexual violence. Only 17(2.6%) of mothers reported as sexually violated. In addition to this, there were no mothers reporting any form of detention in the health care institutions, see Fig. 2 below, and see Table 3 at the end of the document for more information.

**Table 3:** The Magnitude of Obstetric Violence During Labour and Delivery by Domain, Gedeo Zone South Ethiopia, 2020

Category and types of obstetric violence	Answer	
	Yes (%)	No (%)
Physical abuse	147 (22.2%)	514 (77.8%)
Health provider(s) physically hit or slapped	113 (17.1%)	548 (82.9%)
Separate mother from baby without medical indication	28 (4.2%)	633 (95.8%)
Receiving unnecessary Pain-relief treatment	16 (2.4%)	645 (97.6%)
Denied from food or fluid in labour unless medically necessitated	4 (0.6%)	657 (99.4%)
Non-confidential care	152 (22.9%)	509 (77.1%)
The providers didn.t uses drapes or covering to protect mother’s privacy	91 (13.8%)	570 (86.2%)
Health providers discussed your private health information in a way that others could hear	65 (9.8%)	596 (90.2%)
Non - consent care	436 (66%)	225 (34%)
The provider didn’t introduce themselves and greeting mother and her support person	345 (52.2%)	316 (47.8%)
The providers didn’t encourage mother to ask questions	123 (18.6%)	538 (81.4%)
The provider didn’t respond mother’s question with politeness	98 (14.8%)	563 (85.2%)
The provider didn’t explain what is being done and what to expect throughout labour and birth	76 (11.6%)	585 (88.4%)
Provider didn’t give periodic updates on status and Progress of your labour	132 (20%)	529 (80%)
Providers didn’t permit mother to choose of position for	354 (53.5%)	307 (46.5%)
Mother’s Lack of information Obtains consent	361 (54.6%)	300 (45.4%)
Non- dignified care	268 (40.5%)	393 (59.5%)
Health providers shouted at or scolded you	137 (20.7%)	524 (79.3%)
Health providers made negative comments about you	56 (8.5%)	605 (91.5%)
Support staffs insult me and my companion	13 (2%)	648 (98%)
Demonstrating caring culturally in appropriate way	199 (30.1%)	462 (69.9%)
Abandonment/neglect of care	51 (7.7%)	610 (92.3%)
Health providers ignored you when you called for help	41 (6.2%)	620 (93.8%)
Left unattended during the second stage of labor	27 (4.1%)	634 (95.9%)
Discrimination	19 (2.9%)	642 (97.1%)
Health care providers discriminated by race, ethnicity and economic status	16 (2.4%)	645 (97.6%)
Health care providers discriminated because of teenage	8 (1.2%)	653 (98.8%)
Health care providers discriminated because of being HIV positive	–	661 (100%)
Detention in health facility		
Discharge postponed until hospital bills are paid	–	661 (100%)
The woman is either detained or confined her well	–	661 (100%)
Sexual violence	17 (2.6%)	644 (97.4%)
The care provider frequently performs vaginal examination without my intention	14 (2.1%)	647 (97.9%)
The care provider touched me while I have been refusing to touch me	9 (1.4%)	652 (98.6%)

### Factors associated with obstetric violence

#### Bivariate logistic regression analysis of obstetric violence

According to bivariate logistic regression, those variables which have *P*-value ≤0.25 were regressed against the dependent variable in multivariable logistic regression.

In bivariate logistic regression analysis; mother’s educational status, residence, age, religion, parity, occupation, family monthly income of respondent, utilization of ANC, health facility stay after delivery, mode of delivery, time of birth, sex of attendant, type of health facility and mother facing complication during delivery were the candidate variables for multivariable logistic regression. See Table 4 at the end of the document for more information.

**Table 4:** Result of bivariate logistic regression analysis of the candidate variables with obstetric violation among women in Gedio zone south Ethiopia,2020

Variables	Obstetric violence		COR (95%CI)
	Yes	No	
**Age of the mother**			
< 35	449 (81%)	106 (19%)	1.520 (1.016, 2.51) *
≥ 35	78 (73.6%)	28 (26.4%)	1
**Residence**			
Urban	332 (80.2%)	82 (19.8%)	1.0797 (1.19,2.88) **
Rural	195 (78.9%)	52 (21.1%)	1
**Religion**			
Christian	406 (79.8%)	103 (20.2%)	1.0987 (0.09,0.036) **
Muslim	121 (79.6%)	31 (20.4%)	1
**Total births**			
One	134 (84.8%)	24 (15.2%)	1
2–3	278 (79.2%)	73 (20.8%)	0.682 (0.39, 0.067) **
4 and above	115 (75.7%)	37 (24.3%)	0.5567 (0.19, 0.79) **
**Educational status**			
No formal education	174 (72.8%)	65 (27.2%)	1
Primary school	95 (81.2%)	22 (18.8%)	1.6131 (1.15,2.34) *
Secondary school and above	258 (84.6%)	47 (15.4%)	2.0506((1.29,3.26) *
**Occupation**			
Housewife	212 (73.6%)	76 (26.4%)	1
Governmental Employed	131 (87.3%)	19 (12.7%)	2.4717((1.80,4.25) *
Other	184 (82.5%)	39 (17.5%)	1.6913 (1.25,2.31) *
**Mode of delivery**			
AVD	187 (83.1%)	38 (16.9%)	1.389 (1.16–2.789) *
SVD	340 (78%)	96 (22%)	**1**
**ANC utilization**			
Yes	381 (83.2%)	77 (16.8%)	1.9314 (1.23–2.67) *
No	146 (72%)	57 (28%)	**1**
**Monthly income**			
≤ 2500	291 (82%)	64 (18%)	1.348 (1.107–2.52) *
> 2500	236 (77.1%)	70 (22.9%)	**1**
**Staying duration**			
1–2 days	428 (76.2%)	134 (23.8%)	1
3 and above days	81 (81.8%)	18 (18.2%)	0.7098 (0.38, 0.82) *
**Time of birth**			
Day	211 (76.7%)	64 (23.3%)	1
Night	316 (81.9%)	70 (18.1%)	0.7303 (0.42, 1.18) **
**Sex of attendant**			
Female	369 (80.6%)	89 (19.4%)	1.1808 (0.526,3.846) **
Male	158 (77.8%)	45 (22.2%)	1
**Facing complication**			
Yes	182 (89.7%)	21 (10.3%)	2.8386((1.90,4.16) *
No	345 (75.3%)	113 (24.7%)	1
**Type of facility**			
Public	474 (80.9%)	112 (19.1%)	1.7567 (1.25,2.58) **
Private	53 (70.7%)	22 (29.3%)	1

*CI* Confidence interval, *COR* Crude Odds Ratio, *ANC* Antenatal Care, *SVD* Spontaneous Vaginal Delivery, *AVD* Assisted Vaginal Delivery, **1** – reference variable, * - significant at *P* < 0.01, ** - significant at *P* < 0.05

#### Multivariable logistic regression analysis of obstetric violence

In multivariable logistic regression, a total of 14 variables which have *P*-value ≤0.25 during bivariate logistic regression analysis were regressed against the dependent variable. However, in the multivariable logistic regression analysis; utilization of ANC, educational status of mothers, facing complications during labour, and length of stay in the health facility were significantly associated with obstetric violence as see in Table 5.

**Table 5:** results of multivariable logistic regression of factors associated with obstetric violation among women in Gedio zone, south Ethiopia, 2020

Variables	Obstetric violence		AOR (95% CI)	***p***-value
	Yes	No		
**Educational status**				
No formal education	174 (72.8%)	65 (27.2%)	1	
Primary school	95 (81.2%)	22 (18.8%)	1.4131 (1.75,2.94)	0.02
Secondary school and above	258 (84.6%)	47 (15.4%)	2.2573((1.44,3.54)	0.01
**ANC utilization**				
Yes	381 (83.2%)	77 (16.8%)	2.365 (1.62–3.21)	0.003
No	146 (72%)	57 (28%)	1	
**Facing complication**				
Yes	182 (89.7%)	21 (10.3%)	3.1382((2.34,5.17)	0.002
No	345 (75.3%)	113 (24.7%)	1	
**Staying duration**				
1–2 days	428 (76.2%)	134 (23.8%)	1	
3 and above days	81 (81.8%)	18 (18.2%)	0.5367 (0.28, 0.86)	0.003

*CI* Confidence interval, *AOR* Adjusted Odds Ratio, *ANC* Antenatal Care, 1 – reference variable

Mothers who attained secondary school and above were more likely to face obstetric violence as compared to those with no formal education. [AOR =2.2573, (95% CI (1.44,3.54))].

Mothers who do have ANC follow-up were two times more likely to experience obstetric violence as compared to their counterparts [AOR = 2.365 (95%CI, (1.62–3.21))].

Mothers who faced complications during labour were three times more likely to experience obstetric violence. [AOR = 3.1382(95% CI (2.34,5.17))].

Mothers who stay 3 and above days longer are 47% more likely to experience obstetric violence as compared to those who stayed less than 2 days [AOR = 3.1382 (95%CI, ((2.34,5.17))].

### Qualitative study result

#### In- depth interview

The data from the interviews was transcribed and coded using open code software version 4.03. The goal of this study approach was to investigate the obstetric violence experiences of women, community leaders, and obstetric care providers to support a quantitative finding. The following broad data-generating question was used to begin the interview: (1) Are you familiar with obstetric violence? (2) What types of obstetric violence are the most common? (3) Does any of your neighbors or relatives tell you about their violent experiences? (4) Do you have any experience with obstetrical violence? (5) What is the most common cause of obstetric violence? Do you have any experience related to obstetrical violence? The majority of the women polled expressed dissatisfaction with their labor and delivery care. In general, the responses are divided into three categories: mothers’ responses, obstetric care providers’ responses, and community leaders’ responses*.*

#### Mothers’ responses

According to the in-depth interviews with mothers, most respondents experienced providing obstetric care in a culturally inappropriate manner, insulting slapping of the mother during delivery, and not taking consent. The main finding of this study was that the most common form of obstetric violence was non-dignified and non-consented care. This may cause a woman to prefer home delivery rather than hospital care, this in turn leads to a great increase in maternal morbidity and mortality.

*According to one maternity care user, “during my stay in the health facility (primary hospital), the obstetric care providers approach was completely out of my culture.” The care providers helped me in a manner which is convenient form him in contrary to my culture.*

The main findings of this study revealed that delays in the process of written referral from health centres to hospitals cause dissatisfaction with the provision of appropriate maternity care, and it is one of the three delays that lead to maternal morbidity and mortality. According to the in-depth interviews with mothers, the majority of mothers experienced delays in receiving services. Delays in receiving services occur at various stages of the health centre referral process.

*According to a 36-year-old multigravida woman, “I gave birth in a referral hospital, and the client flow was extremely high; I gave birth in the waiting room because there were only three delivery coaches. All maternity care users and health care providers were watching me while I was giving birth in a waiting room. They exposed my privacy to all strangers to watch me. Starting from that day, I fill disappointed on the labor and delivery service of hospitals”.*

The findings of an interview with maternity care users revealed that health care providers’ poor communication with mothers, birth companions during labor and delivery, and providing services first by relatives and friends were challenges raised by the mothers. According to the findings above, discrimination based on certain attributes is common, causing maternity service users to be unsatisfied with healthcare providers. This violates mothers’ rights to fairly and equally childbearing care, regardless of religion, race, or economic status.

#### Community leaders’ response

*Community leaders stated; “In the health facility, some of the obstetric care providers provide care based on the relationship status. They put their relatives and friends first. This makes me disappointed the service being given”.*

The results of the interview with a community leaders found that health care providers‟ poor communication with mothers, birth companions during labour and delivery, and providing services first by relatives and friends were challenges raise by community leaders. Based on the above finding, discrimination based on specific attributes is common, so this makes the maternity service users being dissatisfied by health service care providers. This violates the right of mothers to get equal and unbiased service irrespective of religion, race, and economical status during child bearing.

The result of in-depth interview showed that almost all health care providers from health centres and hospitals reported that one of the most important factors that cause to compromised service and finally leads to obstetric were lack of satisfaction of health care providers related to burnout, payment, and lack of educational opportunity.

#### Obstetric care providers responses

*According to one obstetric care provider, “the main reason that maternity care users complained was the scarcity of infrastructure in health institutions, such as a lack of enough waiting rooms and delivery coaches. This is a common problem in health care facilities with a high case flow, making it difficult to provide optimal maternity care. There are also critical problems in health centres, such as a lack of light and even water”.*

*Obstetric care provider stated; “Some health care providers may be insulting service users, it may be due to work over load especially in the referral hospital, there is high client flow, when we attend more than 300 deliveries per month. We may be exhausted during this time; we may insult maternity care users either intentionally or unintentionally‟.*

From in-depth interview, both obstetric care providers and maternity care provider perceived that insult and pinch are normal during labour and delivery, demoralization of health care provider due week health system, and lack of training were other challenges raised by health care provider.

*Obstetric care provider stated: “I have 5- service years in the health centre with a diploma midwife. There is no growth in salary as well as my educational status. This makes me even to dislike my profession at all.*

The main finding of the analysis was normalization of violent care during labour and delivery.

*Obstetric care provider from the health centre stated; “there is no regular support from our higher officials rather than asking for a monthly report. We also not enrolled in long as well as short-term training‟.*

## Discussion

### Magnitude of obstetric violence

The result showed that 79.7% (527) of mothers experienced obstetric violence with 95% CI (76.9–82.8), this indicates that only 20.3% of mothers were not experienced with obstetric violence, despite the fact of WHO’s statement concerning every woman has to be treated in humanistic and respectfully. In addition to this, as OV is declared as a breach of human right in different countries and it is accepted by WHO, the magnitude of OV in this study indicates that majority of child-bearing women experienced the breach of human right [1, 5]. This may lead to a decrease in health facility child births and results in maternal as well as neonatal morbidity and mortality to rise sharply as an obstetrical violated woman may prefer home delivery with the fear of this type of violated care by obstetric care providers. This study was in line with the study conducted in Amhara Region, Northwest Ethiopia, and Addis Ababa where the overall magnitude of obstetric violence was 75.1 and 78% respectively [25, 26].

The present study is higher than studies conducted in Spain where 44% of child-bearing women reported that they had unnecessary and/or painful procedures during current child birth. The discrepancy is due to the fact that the previous study in Spain was intended on “Interventionism and medicalization during birth” which a particular form obstetric violence [16]. The finding is also higher than the study conducted in Brazil where 12.6% of mothers experienced violence during labour and deliver [27]. The difference may be explained by the socioeconomic background of participants; educational status could affect the magnitude of OV where in the study conducted in Brazil about 77.4% had higher education than present study in which only 22.3% had higher education. As the educational status of the participants became higher and then it is obvious that knowledge regarding the nature of obstetric violence also increase [28, 29].

Higher than the previous community-based studies conducted in Kenya and Tigray, Ethiopia, in which 20 and 22% of mothers experienced obstetric violence respectively [30, 31]*.* This is probably due to the time of the study conducted; because as the term obstetric violence is newly recognized as a violation of human rights, mothers may not report it as a violation of their rights rather consider it as standard obstetric care [27], but currently OV is becoming the top concern in different countries regarding empowering child bearing women towards refusing violent care. This help them to identify the normalized but violent care as a violation of human right [5, 6, 29] The current study used the new advanced tool with revised parameters regarding the components of obstetric violence that may result in a high magnitude of OV than the study conducted previously in Kenya and Tigray region.

This study is slightly lower than the research conducted in Jimma, Ethiopia, where 91.7% of mothers experienced obstetric violence. It is also lower than the study conducted in low-income countries where the magnitude of obstetric violence was 98% [32, 33]. The discrepancy may be explained by setting of the study and the research parameters investigated.

The most commonly reported form of obstetric violence in this study was non-consented care where 66% of mothers reported followed by non-dignified care in which 40.5% of participants reported. The result was also consistent with in-depth interview of maternity care users which showed that non-consented care during the procedure in the maternity care units was common. This result was in line with the study conducted in Amhara, Ethiopia, and Ghanaian where 63.6 and 54.5% of mothers experienced this form of obstetric violence respectively [25, 34]. The common criterion of OV under the category of non-dignified care was a culturally inappropriate manner, with 30.1% of mothers experiencing this specific form of obstetric violence. The finding was lower than the study conducted in Jimma, Ethiopia, in which 75.2% were not given the care in a culturally appropriate way by the care providers [32]. This may also be explained by socio-cultural differences, the study setting, time of the study and the design used.

In addition, the other category of obstetric violence of women experienced during facility-based child birth in this study was physical abuse in which 36% of mothers reported physical abuse during facility child birth. The result was also supported by the qualitative approach in which obstetric care users stated that “health care providers slapped and pinched them during labor and delivery”. This finding is similar to the study conducted in Ghanaian where 35.7% of mothers reported this type of obstetric violence [34]. However, it is different from the study conducted in Tanzania in which only 15% of mothers experienced this form of obstetric violence [35]. This inconsistency might be due to the policy of health care and implemented program differences. The finding on this category of obstetric violence is also lower than the study conducted in Jimma, Ethiopia, where 87.9% of women were not protected from physical harm or ill-treatment during labor and delivery [32]. This may be explained by the small sample size in the previous study and setting of the study in which the study conducted in Jimma was institution-based.

According to this finding, the other category of obstetric violence experienced by women were non –confidential care where 22.59% of mothers reported this form of obstetric violence. This result is slightly lower than the study conducted in Amhara region, Ethiopia, where more than one-third of the mothers experienced this form obstetric violence [25]. It probably due to the obstetric characteristics of the study participants; in the current study mothers with a history of previous caesarean section were excluded since it may affect the magnitude of the problem because this type of women may not remember the type of care she received during the procedure as the effect of anaesthesia does not let her to do so.

The least commonly reported form of obstetric violence in this study is sexual violence in which 2.6% of mothers complained frequent vaginal examinations without the consent of the mother and touching of the labouring mothers’ breast without any medical indications. This result is also supported by in-depth interviews of mothers.

*Obstetric care users stated: “the male obstetric care provider frequently touches my breast while I was refusing to do so, he also frequently examines my birth canal by putting one of his hands in my breast. During that time, I confuse with his actions and I told him that if you touch like that, I will shout and call your colleagues and finally he runs out the room and another female attendant helped me”.*

The result is consistent with the qualitative study conducted in Sri Lanka where women experienced unusual touching (one of his hands was on her breast) by a male obstetric care provider [36].

### Factors associated with obstetric violence

The association of the respondent’s socio-demographic characteristics, obstetric characteristic, and service-related factors with experience of obstetric violence during facility-based delivery was investigated. According to this study, educational status, ANC utilization, duration of stay in health facility, and facing complication during labor and delivery had significant association with obstetric violence during facility-based childbirth.

Secondary and higher educational level was significantly associated with obstetric violence. Similar findings with the study in the findings was also in line with the study conducted in brazil, Tigray and Amhara region [24, 37]. This is may be due to the fact that as a level of education increases, the probability of mothers being aware about the problem (obstetric violence) would also increases. In addition to this, as a level education of mothers gets advanced, they become more resistant to accept violent action during labor and delivery as a normal procedure. So that they become confident enough to state a violent action as a violent [27].

Having ANC during pregnancy visits was significantly associated with obstetric violence. This means mothers who have a history of ANC visits for the recent delivery are more likely to report obstetric violence during labor and delivery than those who don’t have any ANC follow-up. This might be due to when mothers had ANC follow-up, they might get the chance to trace the different types of violences that a woman might face.

In addition to this, other obstetrics factor associated obstetric violence in this study was length of stay in the health facilities. Those mothers who stayed more than 48 hrs are more likely to experience obstetric violence than others. This study was in line with the study conducted in the Tanzania [38]. This might be due to the longer the women stayed at the facility, the higher they will get the chance to be seen by the health care providers.

In this study, in-depth interviews of maternity care users and community leaders showed that accepting obstetric violence as a normal obstetric procedure was a common factor for the prevalence of obstetric violence. Lack of positive attitude of obstetric care providers to wards mothers and his job were factors for obstetric violence during labour and delivery. This may be due to the nature of the problem which is not recognized as a violation human rights among the majority of the international community because it the newly emerging term [1]. This study finding was similar to study conducted in Kenya [30]. The qualitative study conducted in South America also had a similar finding with the current study [29].

Lack of professional development opportunities and provider distancing as a result of lack of training were other challenges which was raised by most of the obstetric care providers in both health centres and hospitals during in-depth interviews. This study is similar to the observational study conducted in five East and South Africa countries in which to luck f resources, staff shortage, and lack of obstetric care providers” training was identified as factors associated with obstetric violence revealed [33, 39]. It also supported by with the study conducted in south America [29].

### Limitations of the study

Self-reporting bias by participants and health care providers, lack of knowledge on OV among both the participants and the HCP, and lack of a validated tools to assess OV was found as the limitation of the study however possible strategies were done to reduce its effect employed.

## Conclusion and recommendations

The finding revealed that the magnitude of obstetric violence during labour and delivery is high (79.7%) in Gedeo zone which is a common issue that needs urgent intervention. The most commonly reported type of obstetric violence is non-consented care in which 66% of mothers reported. The second commonly reported type of obstetric violence as non-dignified care in which 40.5% of participants was reported. Utilization of ANC, educational status of mothers, facing complications during labour, and length of stay in a health facilities were significantly associated variables with obstetric violence. Non dignified care and non-consented care were the most common form of obstetric violence which may lead a woman to choose for home delivery instead of health facility care, this in turn leads to a great increase in maternal morbidity and mortality as supported by the qualitative approach of the study. Therefore, the Minister of Health, the zone health bureau, and non-governmental organizations (NGOs) should work collaborate, and implement programs to mitigate the inadmissibly high magnitude of obstetric violence during labor and delivery in the zone. It is also preferable to raise awareness in order to increase the number of ANC visits, as well as work on developing training programs to reduce obstetric violence. Obstetric care providers are also expected to provide compassionate and respectful care to all mothers who give birth in a health facility, regardless of their socioeconomic status. Providing culturally sensitive obstetric care to all childbearing mothers. To attract more women to health facilities, to make services more woman ﻿friendly, and to humanize services, adequate emphasis must be placed on the provision of woman-centered care in a respectful and non-abusive manner. Furthermore, facility-based observational research in both private and public health facilities is required to address recall bias and other facility-related factors in the area.

## Data Availability

The datasets generated and /or analyzed during the current study are not publicly available due to preserving participant anonymity but are available from the corresponding author upon request through the email address (wondwosenm955@gmail.com).

## Abbreviations

AOR: Adjusted odds ratio
OR: Odds ratio
OV: Obstetric violence
PPS: Probability proportional to size
SES: Socio economic status
SNNPR: South Nations Nationalities and Peoples Region
SPSS: Statistical Package for the Social Sciences
SRS: Simple random sampling
UNICEF: United Nations Children’s (Emergency) Fund
USA: United States of America
USAID: United States Agency for International Development
WHO: World Health Organization

## References

[1] Simonovic D, Women U, Secretary-General U. A human rights-based approach to mistreatment and violence against women in reproductive health services with a focus on childbirth and obstetric violence: note / by the Secretary-General, UN; 2019. Retrieved from https://policycommons.net/artifacts/127145/a-human-rights-based-approach-to-mistreatment-and-violence-against-women-in-reproductive-health-services-with-a-focus-on-childbirth-and-obstetric-violence/ on 13 Jul 2022. CID: 20.500.12592/j9q0j3.

[2] da Silva Carvalho I, De Brito RS. Formas de violência obstétrica vivenciadas por puérperas que tiveram parto normal. Enfermería Global. 2017;16(3):71–97.

[3] Williams CR, Jerez C, Klein K, Correa M, Belizan J, Cormick G (2018). Obstetric violence: a Latin American legal response to mistreatment during childbirth.

[4] Reuther, Marie Luise. “Prevalence of Obstetric Violence in Europe: Exploring Associations with trust, and care-Seeking Intention.” Bachelor's thesis, University of Twente, 2021.

[5] D'Gregorio RP (2010). Obstetric violence: a new legal term introduced in Venezuela. No longer published by Elsevier.

[6] Bellón SS (2014). Obstetric violence: medicalization, authority abuse and sexism within Spanish obstetric assistance. A new name for old issues?.

[7] Chadwick RJ, Cooper D, Harries J (2014). Narratives of distress about birth in south African public maternity settings: a qualitative study. Midwifery.

[8] Chadwick R (2017). Ambiguous subjects: obstetric violence, assemblage and South African birth narratives. Fem Psychol.

[9] Katz L, Amorim MM, Giordano JC, Bastos MH, Brilhante AVM (2020). Who is afraid of obstetric violence?. Revista Brasileira de Saúde Materno Infantil.

[10] Vacaflor CH (2016). Obstetric violence: a new framework for identifying challenges to maternal healthcare in Argentina. Reprod Health Matters.

[11] Mena-Tudela D, Iglesias-Casás S, González-Chordá VM, Cervera-Gasch Á, Andreu-Pejó L, Valero-Chilleron MJ (2020). Obstetric violence in Spain (part I): Women’s perception and Interterritorial differences. Int J Environ Res Public Health.

[12] Bohren MA, Vogel JP, Hunter EC, Lutsiv O, Makh SK, Souza JP (2015). The mistreatment of women during childbirth in health facilities globally: a mixed-methods systematic review. Plos Med.

[13] Bourdreux J. Naming‘Obstetric Violence’: Coercion, Bullying, and Intimidation in Non-Evidence Based Childbirth Interventions. The Journal of Motherhood Studies. 2019;15(1):1–4.

[14] Carvalho IDS, De Brito RS (2017). Formas de violência obstétrica vivenciadas por puérperas que tiveram parto normal. Enfermería Global.

[15] Shrivastava S, Sivakami M (2020). Evidence of ‘obstetric violence’in India: an integrative review. J Biosoc Sci.

[16] Mena-Tudela D, Iglesias-Casás S, González-Chordá VM, Cervera-Gasch Á, Andreu-Pejó L, Valero-Chilleron MJ (2021). Obstetric violence in Spain (part II): interventionism and medicalization during birth. Int J Environ Res Public Health.

[17] Mena-Tudela D, Iglesias-Casás S, González-Chordá VM, Valero-Chillerón MJ, Andreu-Pejó L, Cervera-Gasch Á (2021). Obstetric violence in Spain (part III): healthcare professionals, times, and areas. Int J Environ Res Public Health.

[18] Babu BV, Kusuma YS (2017). Violence against women and girls in the sustainable development goals. Health Promot Perspect.

[19] García-Moreno C, Amin A (2016). The sustainable development goals, violence and women’s and children’s health. Bull World Health Organ.

[20] Zureick-Brown S, Newby H, Chou D, Mizoguchi N, Say L, Suzuki E, et al. Understanding global trends in maternal mortality. Int Perspect Sex Reprod Health. 2013;39(1):32–41. 10.1363/3903213 PMC3886625 23584466

[21] Castro A, Savage V (2019). Obstetric violence as reproductive governance in the Dominican Republic. Med Anthropol.

[22] Martínez-Galiano JM, Martinez-Vazquez S, Rodríguez-Almagro J, Hernández-Martinez A. The magnitude of the problem of obstetric violence and its associated factors: A cross-sectional study. Women Birth. 2020;S1871-5192(20):30359-0. 10.1016/j.wombi.2020.10.002 33082123

[23] World Health Organization (2014). The prevention and elimination of disrespect and abuse during facility-based childbirth: WHO statement.

[24] Gebremichael MW, Worku A, Medhanyie AA, Berhane Y (2018). Mothers’ experience of disrespect and abuse during maternity care in northern Ethiopia. Glob Health Action.

[25] Mihret MS (2019). Obstetric violence and its associated factors among postnatal women in a specialized comprehensive hospital, Amhara Region, Northwest Ethiopia. BMC Res Notes.

[26] Kitaw M, Tessema M. Respectful maternity care and associated factors among mothers in the immediate post–partum period, in public health facilities of Addis Ababa. Int J Pregnancy Child Birth. 2019;5(1):10–7.

[27] Lansky S, Souza KV, Peixoto ERM, Oliveira BJ, Diniz CSG, Vieira NF (2019). Obstetric violence: influences of the senses of birth exhibition in pregnant women childbirth experience. Cien Saude Colet.

[28] Chadwick RJ (2016). Obstetric violence in South Africa. SAMJ. S Afr Med J.

[29] Briceño Morales X, Enciso Chaves LV, Yepes Delgado CE (2018). Neither medicine nor health care staff members are violent by nature: obstetric violence from an interactionist perspective. Qual Health Res.

[30] Abuya T, Warren CE, Miller N, Njuki R, Ndwiga C, Maranga A (2015). Exploring the prevalence of disrespect and abuse during childbirth in Kenya. Plos One.

[31] Gebremichael MW, Worku A, Medhanyie AA, Berhane Y (2018). Mothers’ experience of disrespect and abuse during maternity care in northern Ethiopia. Glob Health Action.

[32] Siraj A, Teka W, Hebo H (2019). Prevalence of disrespect and abuse during facility based child birth and associated factors, Jimma University medical center, Southwest Ethiopia. BMC Pregnancy Childbirth.

[33] Okafor II, Ugwu EO, Obi SN (2015). Disrespect and abuse during facility-based childbirth in a low-income country. Int J Gynaecol Obstet.

[34] Dzomeku VM, van Wyk B, Lori JR (2017). Experiences of women receiving childbirth care from public health facilities in Kumasi, Ghana. Midwifery.

[35] Lukasse M, Schei B, Ryding EL, Bidens Study G (2014). Prevalence and associated factors of fear of childbirth in six European countries. Sex Reprod Healthc.

[36] Perera D, Lund R, Swahnberg K, Schei B, Infanti JJ (2018). When helpers hurt’: women’s and midwives’ stories of obstetric violence in state health institutions, Colombo district, Sri Lanka. BMC Pregnancy Childbirth.

[37] Mihret MS (2019). Obstetric violence and its associated factors among postnatal women in a specialized comprehensive hospital, Amhara Region, Northwest Ethiopia. BMC Res Notes.

[38] Sando D, Ratcliffe H, McDonald K, Spiegelman D, Lyatuu G, Mwanyika-Sando M (2016). The prevalence of disrespect and abuse during facility-based childbirth in urban Tanzania. BMC Pregnancy Childbirth.

[39] Storeng KT, Murray SF, Akoum MS, Ouattara F, Filippi V (2010). Beyond body counts: a qualitative study of lives and loss in Burkina Faso after ‘near-miss’ obstetric complications. Soc Sci Med.

